# Improving Recognition and Weight Monitoring in Children and Adolescents Presenting with Restricted Eating

**DOI:** 10.1192/bjo.2026.11260

**Published:** 2026-06-30

**Authors:** Benjamin Anderson, Sanem Inci Khan, Sharada Deepak, Benjamin Brown

**Affiliations:** Berkshire Health, Reading, United Kingdom

## Abstract

**Aims:**

Improve identification of patients with restricted eating, standardise the assessment and weight monitoring of patients with restricted eating and improve monitoring for at-risk young people.

**Methods:**

This project followed a Plan–Do–Study–Act cycle. The Reading West CAMHS community patient list was analysed to gain a baseline measurement of:

• The consistency in identification of patients with restricted eating.

• The effectiveness of weight monitoring being undertaken for the patients on the team caseload.

An anonymous survey was sent to the multidisciplinary team to analyse perceived barriers to identifying patients with restricted eating and gather perspectives.

A focus group was organised involving clinical psychology, psychotherapy and medical teams to examine these barriers and to generate potential interventions.

Based on these findings, changes were implemented in the team, with the intention to expand to other localities if shown to be effective.

Interventions:

• Development of a standardised protocol for documenting weight.

• Addition of screening questions for restricted eating to the proforma for initial assessments of young people.

• Education for the MDT on the risks associated with restricted eating and the importance of early identification and consistent monitoring.

These interventions were disseminated across the team using established channels.

**Results:**

Of the 49 patients on the treatment list, 19 (39%) had documented evidence of restricted eating. Of these, 7 (37%) had adequate weight monitoring and documentation.

Reviews of the notes of these patients found that weight was not always documented, and if it was it was not in a standardised format.

Survey responses highlighted recurring themes including a lack of training and confidence in identifying restricted eating, concerns about the impact of monitoring on therapeutic relationships and the absence of a local protocol. Additional concerns were raised regarding escalation pathways and clinical responsibility.

**Conclusion:**

The preliminary findings indicate that patients with restricted eating make a significant proportion of the caseload, however monitoring of these patients and documentation is not standardised. This indicates missed opportunities to identify and monitor these at-risk patients.

Several barriers to the identification and efficient weight monitoring of at-risk patients were found. These included staff training and confidence and poor standardisation of practice.

We anticipate improved consistency in weight monitoring and identification of high-risk patients with the implemented changes. Repeat analysis will be needed to confirm the post-intervention effect.

Further work will include the development of robust monitoring protocols, establishment of clear escalation pathways and implementation of defined clinical responses for young people identified as high risk.

